# Evidence-based tests to monitor adults with type 2 diabetes mellitus in primary care: rapid reviews and consensus process

**DOI:** 10.3399/BJGP.2024.0744

**Published:** 2025-09-09

**Authors:** Martha MC Elwenspoek, Rachel O’Donnell, Joni Jackson, Sarah Dawson, Katie Charlwood, Alastair D Hay, Jessica Watson, Penny Whiting

**Affiliations:** 1 National Institute for Health Research Applied Research Collaboration West (NIHR ARC West), University Hospitals Bristol NHS Foundation Trust, Bristol, UK; 2 Population Health Sciences, Bristol Medical School, University of Bristol, Bristol, UK

**Keywords:** blood tests, chronic disease, evidence-based medicine, primary health care, type 2 diabetes mellitus

## Abstract

**Background:**

When monitoring long-term conditions, both over- and undertesting can risk patient harm and increase healthcare costs.

**Aim:**

To evaluate the evidence base for type 2 diabetes mellitus (T2DM) monitoring tests and develop methods for creating evidence-based testing strategies.

**Design and setting:**

Rapid reviews were conducted and a consensus process then used to evaluate the evidence base within primary care settings.

**Method:**

The authors identified tests that are recommended or used commonly to monitor T2DM. Filtering questions were created to examine the rationale for use of each test, which were answered by stepwise rapid reviews of evidence cited by guidelines, systematic reviews, and individual studies. A consensus group of patient representatives and clinicians voted whether tests should be included or excluded based on the evidence or whether further evidence was needed.

**Results:**

Of 15 tests, only haemoglobin A1c, to monitor disease progression and treatment response, and estimated glomerular filtration rate, to detect chronic kidney disease, have a strong evidence base. Based on available evidence and consensus group feedback, routinely testing for fructosamine to monitor disease progression; thyroid function, vitamin B12, ferritin, folate, clotting, bone profile, C-reactive protein, erythrocyte sedimentation rate, and B-type natriuretic peptide; and liver function for adverse treatment effects of metformin was deemed unnecessary. The study found insufficient evidence for lipids and haemoglobin to screen for secondary conditions, and for vitamin B12 to screen for adverse effects in those taking metformin.

**Conclusion:**

The study found that the evidence base for most T2DM monitoring tests is weak or absent. Clinicians should avoid non-evidence-based tests unless there are additional clinical indications for testing. Standardised evidence-based testing panels for T2DM and other long-term conditions could reduce unnecessary testing.

## How this fits in

Guidelines are unclear about how to optimally monitor long-term conditions. Forty per cent of primary care tests are related to monitoring but which tests are used, and how often, varies substantially across the UK. This study identified a list of tests without strong evidence for the routine monitoring of people with type 2 diabetes mellitus (T2DM). Implementing the study's findings could reduce unnecessary testing for patients with T2DM; this could avoid costly consequences from overtesting, such as follow-up appointments, re-testing, referrals, invasive procedures, increasing clinician workload, and patient harm.

## Introduction

Over 26 million people in the UK have ≥1 long-term condition.^1^ An estimated 4.7 million people in the UK have diabetes; of these, 90% have type 2 diabetes mellitus (T2DM).^2^ It is generally suggested that regular monitoring is beneficial for people with long-term conditions, but evidence for this is lacking. Earlier detection of disease progression or the development of secondary conditions allows for earlier intervention, which may improve patient outcomes. Most long-term conditions in the UK are monitored in primary care, where >40% of all tests ordered are used to monitor existing disease or medications.^3^

To ensure that monitoring improves patient outcomes, the number and frequency of tests need to be optimised because both over- and undertesting can be harmful. Undertesting may lead to delayed diagnoses, complications, patient harm, and litigation. Overtesting leads to an increased number of borderline results^3^ and false-positive results,^4^ which can result in unnecessary and possibly invasive follow-up tests, overdiagnosis, and unnecessary treatment. This cascade effect of medical interventions causes patient harm and anxiety.^5^ Previous research has estimated that 25% of pathology testing may be unnecessary.^3,6^

Currently, long-term condition monitoring is not optimal.^7–10^ Guidelines are unclear about what optimal monitoring looks like and are largely based on expert opinion.^7^ In the absence of clear national guidance, GP practices use local guidance that varies significantly from practice to practice.^8^ This has resulted in great variation in test use across the country.^9,10^ To avoid under- and overtesting, a good rationale is needed for each monitoring test underpinned by strong evidence. The aim was to develop a methodology to evaluate the rationale of monitoring tests and identify the evidence base for this rationale. This methodology was applied to blood tests that are currently used to monitor people with T2DM in primary care to highlight the gaps in the evidence base and identify a ‘minimal’ testing panel required for all patients with T2DM. This study is part of a larger programme of work, in which the authors aim to develop and test the cost-effectiveness of evidence-based testing strategies including optimal frequency of testing and information resources for patients and clinicians.

## Method

### Identifying candidate tests

The authors created a list of ‘candidate’ tests to consider that were identified through reviewing national guidelines including all tests that are currently recommended,^7^ a survey of clinicians and selecting tests ordered by the majority of the clinicians,^11^ and an analysis of routinely collected UK primary care data, including tests ordered >0.1 test per person per year for patients with T2DM.^9^

Tests were categorised under three reasons for testing: to 1) monitor disease progression and treatment response; 2) screen for secondary conditions; or 3) screen for adverse treatment effects for patients on stable drug treatment (that is, during the maintenance phase of monitoring; monitoring during treatment initiation and titration was not considered). For pragmatic reasons this study limited the investigation to the most common first-line treatments and known side effects listed in the British National Formulary.

An advisory group, including primary and secondary care clinicians and patient representatives, were invited to add (but not remove) tests and verify the categorisation.

### Filtering questions to refine the list of candidate tests

A stepwise list of filtering questions was developed to evaluate the rationale for each test. For tests monitoring 'disease progression' or 'treatment response', these were:

1. Can you intervene to improve treatment response or to slow or prevent further disease progression?
2. Are there clear benefits of earlier detection or treatment?

For tests screening for 'secondary condition' and 'adverse treatment effect', these were:

1. Are people with the long-term condition at an increased risk of developing the secondary condition or side effect that the test is able to detect?
2. Is there anything the clinician can do to manage or treat this?
3. Are there clear benefits of earlier detection or treatment?

### Rapid reviews to answer filtering questions

**Figure 1.**
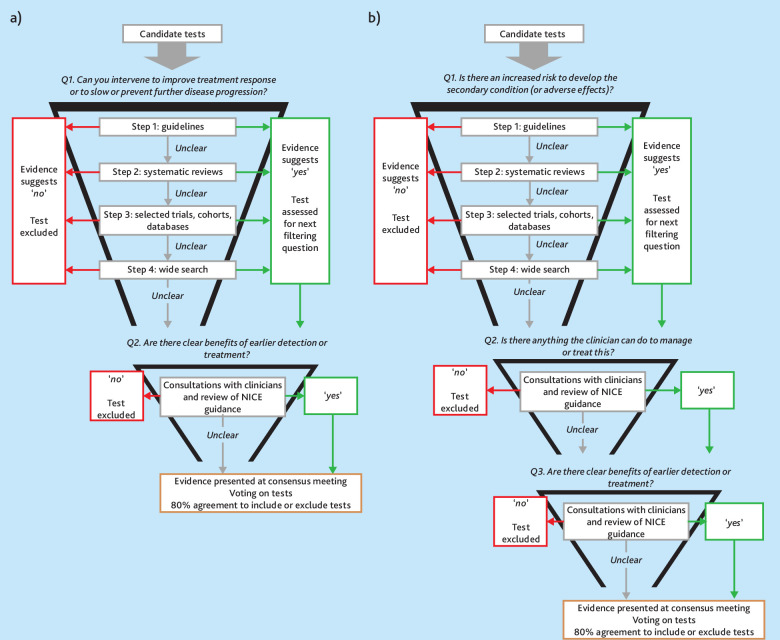
a) Methods to filter tests for monitoring disease progression or treatment response; and b) methods to filter tests for screening for secondary conditions and adverse treatment effects. NICE = National Institute for Health and Care Excellence. UKPDS = UK Prospective Diabetes Study.

The authors conducted rapid reviews to answer each filtering question using a stepwise approach following PRISMA reporting guidelines where appropriate. If strong, up-to-date evidence was identified the authors considered the filtering question answered. The authors moved to the next step if insufficient evidence was identified to answer a filtering question (Figure 1). Owing to the nature and multitude of these rapid reviews they were not registered and separate protocols have not been published. The searches were developed by an information specialist (Supplementary Tables S1–S8 show the full search strategies). Screening was performed in Rayyan.^12^ Data were extracted in standardised extraction forms in Microsoft Excel, which were tested by two reviewers and amended, as necessary, before use. Data extraction and risk of bias (ROB) assessment were performed by one reviewer. ROB was assessed using standardised tools: ROBIS for systematic reviews,^13^ RoB 2 for randomised controlled trials,^14^ and ROBINS-E for cohort studies.^15^

First, the authors identified and appraised the evidence cited by National Institute for Health and Care Excellence (NICE) guidelines. Full texts of identified studies were retrieved, data were extracted, and ROB assessed.

Second, the authors identified and appraised evidence from studies that were part of the UK Prospective Diabetes Study (UKPDS), a pragmatic choice to rapidly identify high-quality evidence. The UKPDS is a landmark randomised, multicentre trial of glycaemic therapies in 5102 patients with newly diagnosed T2DM.^16^ The UKPDS study created a unique cohort with the longest prospective follow-up in a T2DM population.^17^ Manuscripts listed on the UKPDS website (www2.dtu.ox.ac.uk/UKPDS) were screened. All abstracts and full texts were screened independently by two reviewers and conflicts were discussed. Data were extracted on study design, population characteristics, number of included patients, and study results.

Third, the authors identified and appraised evidence from systematic reviews published in English since 2015. The authors searched the KSR Evidence database by combining terms for the long-term conditions and tests, secondary conditions, or side effects. Records were screened independently by two reviewers prioritising recent publications. Once a systematic review of sufficient quality (low ROB) was identified, the authors stopped screening. The inclusion criteria, number of included studies and patients, and summary estimates were extracted. The authors extracted the ROB judgement in KSR Evidence if available (based on the ROBIS tool).^13^

Fourth, the authors identified and appraised evidence from primary studies applying the same eligibility criteria as above, prioritising recent studies in UK populations. MEDLINE, Embase, and CENTRAL were searched, combining search terms for the long-term conditions, tests, secondary conditions, adverse events, and selected cohorts as appropriate. In total, 20% of abstracts and full texts were screened independently by two reviewers and conflicts were discussed. The remaining records were screened by one reviewer. Potentially relevant records were discussed with a second reviewer. Data were extracted on study design, population characteristics, number of included patients, and results for each included study.

### Evidence report

The evidence identified through the rapid reviews was summarised in a report and circulated to an advisory group before a consensus meeting. Evidence was labelled as:

- 'good evidence' if the authors found high-quality systematic reviews or high-quality studies with large sample sizes;
- 'moderate evidence' if the authors found a systematic review with some quality concerns or several primary studies showing similar results but some studies having quality concerns; and
- 'weak evidence' if there was a single primary study with a small sample size with or without quality concerns.

For each test, the filtering questions were answered according to the available evidence as 'yes', 'no', or 'insufficient evidence'.

### Consensus process

At a consensus meeting, the evidence for each test was presented, followed by a vote: 1) include test; 2) exclude test; or 3) insufficient evidence. A consensus of >80% was needed to include or exclude a test. A high consensus threshold was chosen, as this study's recommendations may influence long-term care for patients. If there was no consensus, the evidence was discussed followed by a second vote. If no consensus was reached after the second vote, the test was deemed to need further evidence.

### Post-consensus meeting

One additional rapid review was conducted in response to questions raised at the consensus meeting following the same methods as above (step 4; see Figure 1). The review question was whether the patient knowing the results of lipid or liver function testing can influence patient behaviour and motivate them to implement lifestyle modifications. This was addressed with a series of systematic searches using an iterative approach. MEDLINE and Embase databases were searched.

### Patient and public involvement

Two patient representatives reviewed the funding application and commented on the plain language summary. During the project, they actively participated in monthly project meetings as part of the project management group. They advised during the recruitment of further patient representatives. Four patient representatives contributed to the consensus meeting, where they had equal voting rights to the clinicians.

## Results

### Candidate tests

A total of 15 candidate tests were identified. Answers to the filtering questions are summarised in Tables 1 [Table table2]-3 and evidence for each test is provided in more detail in Supplementary Tables S9–S11. See Supplementary Figures S1–S4 for PRISMA flow diagrams of each rapid review.

**Table 1.** Tests to monitor disease progression and treatment response — answers to filtering questions

Can you intervene to improve treatment response?	Benefits of earlier detection?	Which test should be used to monitor diabetic control?	Consensus meeting outcome
Yes	Yes	HbA1c (good evidence)	85% (*n* = 11/13) consensus to include HbA1c
Yes	Yes	Not fructosamine	92% (*n* = 12/13) consensus to exclude fructosamine

HbA1c = haemoglobin A1c.

**Table 2. table2:** Tests to screen for secondary conditions — answers to filtering questions

Test	Reason for testing	Are people with T2DM at risk?	Can you intervene?	Benefits of earlier detection?	Consensus meeting outcome	Offer routinely to everyone with T2DM?	Evidence gaps/comments
Renal function tests (eGFR)	To detect renal disease	Yes (good evidence)	Yes	Yes (good evidence)	92% (*n* = 12/13) consensus to include	Yes	Optimal frequency of testing
Liver function tests	To detect non-alcoholic fatty liver disease	Yes (good evidence)	No (only lifestyle advice)	Unclear (no evidence)	No consensus	No	The advisory board questioned whether testing improves people’s response to lifestyle advice. The evidence base on this is inconsistent and weak
Lipid profile	To detect dyslipidaemia and assess cardiovascular risk	Yes (good evidence)	Yes	Unclear (CVD risk can be calculated without lipids)	No consensus	Unclear	Lipid profile testing is carried out at T2DM diagnosis, and the advisory board questioned whether further monitoring is beneficial. What is the benefit of including lipids in the QRISK score?
Haemoglobin (full blood count)	To detect anaemia	Yes (moderate evidence)	Yes	Yes (weak evidence)	No consensus	Unclear	The advisory group requested additional evidence on the incidence of anaemia in patients with T2DM
Thyroid function tests	To detect hyper- or hypothyroidism	No (moderate evidence)	Yes	Unclear	85% (*n* = 11/13) consensus to exclude	No	—
Vitamin B12 tests	To detect deficiencies and anaemia	Unclear (weak, inconsistent evidence)	Yes	Unclear	85% (*n* = 11/13) consensus to exclude	No	Increased risk of vitamin B12 deficiency in patients with T2DM is most likely owing to metformin, not the condition itself
Ferritin tests	To detect deficiencies and anaemia	Yes (good evidence)	Yes	Unclear	85% (*n* = 11/13) consensus to exclude	No	The advisory group found the test too non-specific to inform clinical management
Folate tests	To detect deficiencies and anaemia	Unclear (weak, inconsistent evidence)	Yes	Unclear	85% (*n* = 11/13) consensus to exclude	No	—
Clotting tests	To detect bleeding or clotting disorders	Unclear (no evidence)	Yes	Unclear	92% (*n* = 12/13) consensus to exclude	No	The advisory group agreed that there is no reason to suspect more clotting disorders in people with T2DM
Bone profile	To detect bone cancers, vitamin D deficiency, or parathyroid problems	No (moderate–good evidence)	Yes	Unclear	100% (*n* = 13/13) consensus to exclude	No	—
C-reactive protein and erythrocyte sedimentation rate	To detect inflammation	Yes (good evidence)	No (only lifestyle advice)	Unclear	100% (*n* = 13/13) consensus to exclude	No	—
B-type natriuretic peptide	To detect heart failure	Yes (good evidence)	Yes	Unclear (no evidence)	100% (*n* = 13/13) consensus to exclude	No	The advisory group noted that the test has low accuracy for heart failure and was not developed as a monitoring test

CVD = cardiovascular disease. eGFR = estimated glomerular filtration rate. T2DM = type 2 diabetes mellitus.

**Table 3.** Tests to screen for adverse treatment effects of metformin for patients on stable treatment — answers to filtering questions

Test	Are people with T2DM at risk?	Can you intervene?	Benefits of earlier detection?	Consensus meeting outcome	Offer routinely to everyone on metformin?	Evidence gaps
Renal function test	No (moderate evidence)	No	Unclear	No consensus	No	The advisory group noted that metformin is contraindicated for CKD4, but that is screening for a secondary condition, which has been covered by renal function tests (eGFR) screening for CKD in Table 2
Liver function tests	No (moderate evidence)	No	Unclear	92% (*n* = 12/13) consensus to exclude	No	—
Vitamin B12	Yes (good evidence)	Yes	Unclear (no evidence)	No consensus	Unclear	The risk of vitamin B12 deficiency increases with length of use and increased dosage, so it is unclear whether screening everyone from the start is necessary. It is unclear whether there is a clear benefit to detect B12 deficiency before the onset of symptoms

CKD = chronic kidney disease. CKD4 = stage 4 CKD. eGFR = estimated glomerular filtration rate. T2DM = type 2 diabetes mellitus.

### Tests with strong evidence and rationale

Only HbA1c and eGFR, out of the 15 tests considered, had strong evidence (Tables 1 and 2).

### Tests with unclear evidence or rationale

There was also insufficient evidence to justify routine liver function tests to detect non-alcoholic fatty liver disease, lipid tests for dyslipidaemia or cardiovascular risk, haemoglobin for anaemia, or vitamin B12 for metformin-related adverse effects. There was insufficient evidence on whether testing influences lifestyle changes. The effect may go in either direction (for example, a normal test result may increase or decrease the likelihood of behavioural change), so current evidence does not support the use of monitoring tests solely to promote behavioural change (Tables 2 and 3).

### Tests that should not be routinely offered to patients with T2DM

Based on the evidence and a consensus meeting with an advisory group, 11 tests were identified that should not be routinely offered to people with T2DM. Fructosamine cannot replace HbA1c to monitor disease progression and should only be offered on a patient-by-patient basis if HbA1c cannot give correct results (because of genetic variant of haemoglobin). There is no rationale to offer thyroid function tests and bone profile tests, because evidence shows that people with T2DM are not at higher risk of related outcomes than the general population. Similarly, people on metformin are not at greater risk of abnormal renal or liver function, so these should not be offered routinely to people on stable metformin treatment. Also vitamin B12, ferritin tests, folate tests, clotting tests, C-reactive protein (CRP), erythrocyte sedimentation rate (ESR), and B-type natriuretic peptide (BNP) were excluded because of lack of rationale or evidence (Tables 2 and 3).

## Discussion

### Summary

This study found that the evidence base for most blood tests used to monitor T2DM is weak or absent. Having T2DM alone does not indicate the need for thyroid function tests, vitamin B12, ferritin tests, folate tests, clotting tests, bone profile, CRP, ESR, or BNP to screen for secondary conditions. However, if additional indications arise, these tests can and should be used. There is strong evidence supporting the use of HbA1c to monitor disease progression and treatment response. Fructosamine should only be considered if HbA1c cannot give correct results in patients with specific genetic variants. There was insufficient evidence on whether testing influences lifestyle changes. In the absence of strong evidence and the fact that the effect may go in either direction, tests should not be offered for the sole purpose of promoting behavioural change.

### Strengths and limitations

To the authors’ knowledge, this is the first study to attempt to create evidence-based testing panels for long-term conditions. The main strength of this study is the use of a standardised approach and established methods to identify evidence.^18^ The authors of the current study developed new methods to determine the rationale of blood tests for T2DM management that can be used to optimise monitoring of other long-term conditions. Another strength is the involvement of a diverse advisory group that included clinicians and patient representatives. The advisory group helped to interpret the evidence and highlight additional factors to consider that were not captured by the filtering questions.

A weakness of this study is that the authors had to make some compromises in evidence collection for pragmatic reasons. Rapid review methods and a stepwise approach were used to identify evidence rather than full systematic reviews. As such, this study may have missed relevant evidence. However, full-text screening was stopped once strong evidence addressing the filtering question had been identified. Finally, accuracy or the natural variability of each test was not taken into account and optimal frequency of testing or optimal thresholds to use per test were not addressed.

### Comparison with existing literature

This study showed that for eGFR and HbA1c there is a good rationale underpinned by strong evidence to test in people with T2DM and this is in line with current guidelines.^19,20^ Similarly, a study assessing the clinical value and cost-effectiveness of screening programmes for kidney disease using simulation methods concluded that annual monitoring of people with type 2 diabetes to identify the development of early kidney disease was cost-effective and beneficial for patients.^21^

This research suggests that a lot of current testing is unnecessary. For instance, this study showed that there is no rationale to routinely test people with T2DM with liver function or thyroid function tests. However, liver function tests are the third most commonly ordered tests for people with T2DM, with the average patient receiving 1.5 tests per year, and thyroid function tests are the sixth most commonly ordered, with many patients receiving annual testing (0.8 per person per year).^9^

This research identified tests lacking strong evidence that are still routinely used and recommended by guidelines. For example, lipid profile tests are used routinely in patients with T2DM (1.2 tests per person per year),^9^ but increasing evidence suggests that most lipid monitoring is unhelpful. The signal–noise ratio in cholesterol monitoring is weak, which means that frequent monitoring leads to more false-positive than false-negative results. It has been estimated that it takes at least 3 years,^22,23^ or even >5 years,^24^ before the signal exceeds the noise. In other words, it can take at least 3 years before plausible changes in true cholesterol levels exceed analytical and biological variation. In line with this, the minimum difference between two consecutive lipid test results that must be exceeded before the change is considered statistically significant (also known as reference change value) is high: 11%–20% in total cholesterol and 21%–30% in high-density lipoprotein and low-density lipoprotein.^25^

There was also no consensus on whether people on metformin should be tested regularly for vitamin B12 levels, to screen for adverse treatment effects, even though good evidence was found that metformin use increases the risk of B12 deficiency. Since metformin-induced B12 deficiency depends on dosage and length of use, and it takes time to develop B12 deficiency and related issues, it may not be necessary to regularly monitor B12 levels in every patient taking metformin.

### Implications for research and practice

Future research is needed to estimate the optimal frequency of testing, which will be addressed as part of the wider programme of work, which uses economic modelling to estimate the cost-effectiveness of different testing frequencies. Annual testing is often chosen for pragmatic reasons, but these are not evidence based and can therefore lead to adverse consequences related to overtesting.^26,27^ The authors are currently evaluating the clinical and cost-effectiveness of this evidence-based testing panel to monitor T2DM in primary care in a cluster randomised controlled trial ('Test Smart'). Future research should also take the diagnostic accuracy of these routine tests into account and determine the optimal threshold to use, as these are essential parts of a testing strategy.^22^

Clinicians should avoid non-evidence-based tests such as thyroid function tests or bone profile unless there are additional clinical indications for testing. For tests where the evidence is uncertain, the final decision to monitor, what to monitor, and how often should still be made on an individual basis and in discussion with the patient.

Although tests are generally inexpensive, unnecessary testing leads to costly consequences such as follow-up appointments, re-testing, referrals, invasive procedures, increasing clinician workload, and patient harm. The methodology presented here can be applied to other long-term conditions to optimise the use of monitoring tests. Standardising the monitoring of long-term conditions based on the latest evidence can reduce unwanted variation in testing and has the potential to prevent patient harm and increased healthcare costs related to both over- and undertesting.

## Data

The data that support the findings of this study are available within this article and supplementary files.
